# Menin and RNF20 recruitment is associated with dynamic histone modifications that regulate signal transducer and activator of transcription 1 (STAT1)-activated transcription of the interferon regulatory factor 1 gene (*IRF1*)

**DOI:** 10.1186/1756-8935-3-16

**Published:** 2010-09-08

**Authors:** Lauren J Buro, Edmond Chipumuro, Melissa A Henriksen

**Affiliations:** 1 Department of Biology, The University of Virginia, Charlottesville, VA, USA

## Abstract

**Background:**

Signal transducer and activator of transcription (STAT) activation of gene expression is both rapid and transient, and when properly executed it affects growth, differentiation, homeostasis and the immune response, but when dysregulated it contributes to human disease. Transcriptional activation is regulated by alterations to the chromatin template. However, the role of histone modification at gene loci that are activated for transcription in response to STAT signaling is poorly defined.

**Results:**

Using chromatin immunoprecipitation, we profiled several histone modifications during STAT1 activation of the interferon regulatory factor 1 gene (*IRF1*). Methylated lysine histone proteins H3K4me2, H3K4me3, H3K79me3, H3K36me3 and monoubiquitinated histone ubH2B are dynamic and correlate with interferon (IFN)γ induction of STAT1 activity. Chemical inhibition of H3K4 methylation downregulates *IRF1 *transcription and decreases RNA polymerase II (Pol II) occupancy at the *IRF1 *promoter. MEN1, a component of a complex proteins associated with Set1 (COMPASS)-like complex and the hBRE1 component, RNF20, are localized to *IRF1 *in the uninduced state and are further recruited when *IRF1 *is activated. RNAi-mediated depletion of RNF20 lowers both ubH2B and H3K4me3, but surprisingly, upregulates IFNγ induced *IRF1 *transcription. The dynamics of phosphorylation in the C-terminal domain (CTD) of Pol II are disrupted during gene activation as well.

**Conclusions:**

H2B monoubiquitination promotes H3K4 methylation, but the E3 ubiquitin ligase, RNF20, is repressive of inducible transcription at the *IRF1 *gene locus, suggesting that ubH2B can, directly or indirectly, affect Pol II CTD phosphorylation cycling to exert control on ongoing transcription.

## Background

In response to a variety of extracellular ligands, signal transducers and activators of transcription (STATs) are rapidly recruited from their latent state in the cytoplasm to cell surface receptors, where they are phosphorylated by tyrosine kinases. They then translocate to the nucleus, bind DNA response elements and drive the transcription of target genes, affecting growth, differentiation, homeostasis and the immune response [1]. Not surprisingly, given their widespread involvement in normal cellular processes, dysregulation of STAT activity contributes to human disease, particularly to cancers. Persistently active STAT3 and STAT5 are present in breast cancers, head and neck cancers, prostate cancers, multiple myeloma, leukemias and lymphomas [2,3].

STAT activation is both rapid and transient, with the downregulation of STAT activity achieved by several mechanisms, including dephosphorylation by the 45-kDa nuclear phosphatase T cell protein tyrosine phosphatase (TC45), which inactivates the STATs by removing their required tyrosine phosphates, and the cytoplasmic phosphatase Src homology region 2 domain-containing phosphatase-1 (SHP-1), which dephosphorylates the kinases upstream of STATs. Other negative regulators include the suppressor of cytokine signaling (SOCS) family of proteins, which are induced by cytokine signaling and STAT activation and participate in a negative feedback loop, and the protein inhibitor of activated STAT (PIAS) family of proteins, which can directly inhibit STATs by preventing their DNA binding [4].

Transcriptional activation, like that mediated by STATs, is one of several nuclear processes regulated by alterations to the chromatin fiber. Such alterations are dynamic and include covalent histone modifications and DNA methylation, as well as the activities of ATP-dependent complexes [5]. Chromatin's core structure, the nucleosome, is composed of 146 bp of DNA wrapped around an octamer of histone proteins (H3, H4, H2A and H2B). A number of post-translational modifications to the nucleosome, mostly in histone N-terminal tails, have been described, including methylation, acetylation, phosphorylation and ubiquitination. These covalent modifications define the functional state of chromatin via both *cis *and *trans *mechanisms. *Cis *mechanisms, best typified by acetylation/deacetylation, result in changes to nucleosome packing that increase or decrease DNA accessibility. In *trans *mechanisms, non-histone proteins that possess particular binding domains recognize specific histone modifications and recruit additional factors that regulate chromatin structure [6,7]. Together, *cis *and *trans *mechanisms embellish the chromatin fiber to generate biological effects that extend beyond the DNA sequence alone.

Several genome-wide studies have investigated how particular histone methylations correlate with gene expression in human cells [8-14]. Dimethylation and trimethylation of lysine 4 (H3K4me2, H3K4me3) in the N-terminal tail of histone H3 are known to correlate with an active chromatin state. Trimethylation of lysine 27 (H3K27me3) is associated with silenced chromatin, while monomethylation of this same residue is broadly localized to euchromatin. H3K36me3 is enriched downstream of the promoters of actively transcribed genes, and thus, is strongly correlated with H3K4me3, but is not correlated at all with H3K27me3. H3K9me3 is typically associated with transcriptional repression, as is H4K20me1 [15] although others have linked these modifications to the activation of some genes [9,16,17]. H3K79me3 is associated with actively transcribed genes but Barski *et al*. found this modification is enriched at some silent genes [9,18,19]. Such discrepancies suggest that the function of histone methylation might be gene specific and depend upon the recruitment of different trans-acting complexes, or that methylation status might be dynamic and titrated during gene expression by the concerted activity of histone methyltransferases (HMTs) and demethylases (HDMs) [20-23].

In mammalian cells, there are multiple H3K4 methyltransferases, including SET1A/B and mixed lineage leukemia (MLL) proteins 1-4, which contribute to complex proteins associated with Set1 (COMPASS) and COMPASS-like complexes, respectively [24]. WDR82, the human homolog of Cps35/Swd2, is associated only with SET1A/B complexes (not MLL1-4 complexes). Like Cps35, WDR82 interacts with chromatin in a manner that depends upon the monoubiquitination of H2B [25], suggesting a mechanism similar to the crosstalk mechanism that is well established in *Saccharomyces cerevisiae *where H3K4 methylation requires H2B monoubiquitination (ubH2B). Along these same lines, *in vitro *transcription assays suggest that the presence of ubH2B on chromatin stimulates SET1/COMPASS-dependent H3K4 dimethylation and trimethylation [26]. It was reported recently that ubH2B is associated with the transcribed regions of highly expressed genes in human cells. However, RNF20/BRE1A, a human homolog of the yeast E3 ubiquitin ligase, Bre1, can both activate and repress distinct subsets of genes [27,28].

While little is known about the role of histone modification in regulating the activity of genes that are poised for transcription in response to STAT signaling, the rapid and transient nature of STAT-triggered transcription makes it an attractive system in which to study dynamic signaling to chromatin. Interferon (IFN)γ activation of STAT1 drives higher order chromatin remodeling at the major histocompatability complex (MHC) and correlates with recruitment of the chromatin-remodeling enzyme BRG and histone acetylation [29,30]. However, surprisingly, histone deacetylase activity is reportedly required for the transcription of IFN-responsive genes, but the mechanistic basis for this requirement is unknown [31,32].

Here we report the detailed profiling of the changes in the histone modification landscape that accompany STAT1-activated expression of the interferon regulatory factor 1 gene (*IRF1*). To begin to address the molecular basis for these changes, we identified a COMPASS-like complex as the likely H3K4 methyltransferase associated with the *IRF1 *promoter and showed that full H3K4 trimethylation depends upon the activity of the ubiquitination enzyme RNF20/BRE1A. Furthermore, our data indicate that H2B monoubiquitination, located across the *IRF1 *gene, represses transcription when STAT1 signaling is induced by IFNγ.

## Results

### Histone modifications correlate with inducible STAT1 activity

**Figure 1:**
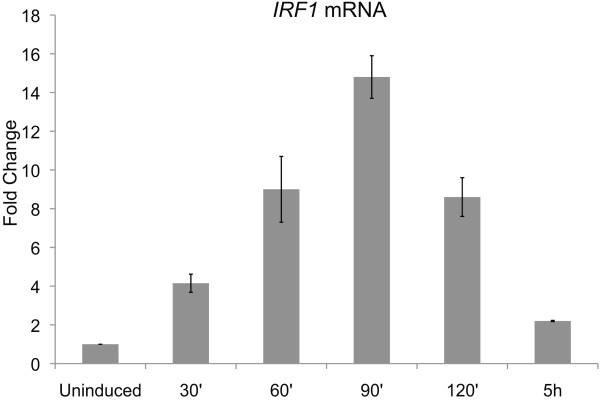
**Time course of interferon regulatory factor 1 gene (*IRF1*) expression in response to interferon (IFN)γ treatment**. 2fTGH cells were treated with IFNγ (5 ng/ml) for 30 min and then returned to normal growth media. Reverse transcription quantitative PCR (RT Q-PCR) was performed after the times shown to quantitate *IRF1 *expression relative to glyceraldehyde 3-phosphate dehydrogenase gene (*GAPDH*) expression, and presented as fold change upon induction. Error bars represent standard error (*n *= 3).

To investigate chromatin's contribution to STAT-induced gene expression, we generated a detailed profile of the distribution of several histone modifications across the *IRF1 *gene locus in 2fTGH cells treated with IFNγ using chromatin immunoprecipitation (ChIP). IFNγ rapidly triggers *IRF1 *gene expression by activating STAT1 homodimers via the Janus kinase (JAK)-STAT signaling pathway. Therefore, a 2fTGH derived cell line, termed U3A, that lacks STAT1 expression was used as a negative control [33]. To assay for dynamic changes in histone modification, we collected ChIP data before, during and after STAT activation, having determined that *IRF1 *gene expression peaked at approximately 90 min and dissipated within 5 h when cells were treated with IFNγ for 30 min and then returned to normal growth media (Figure 1 and Additional file 1e).

**Figure 2:**
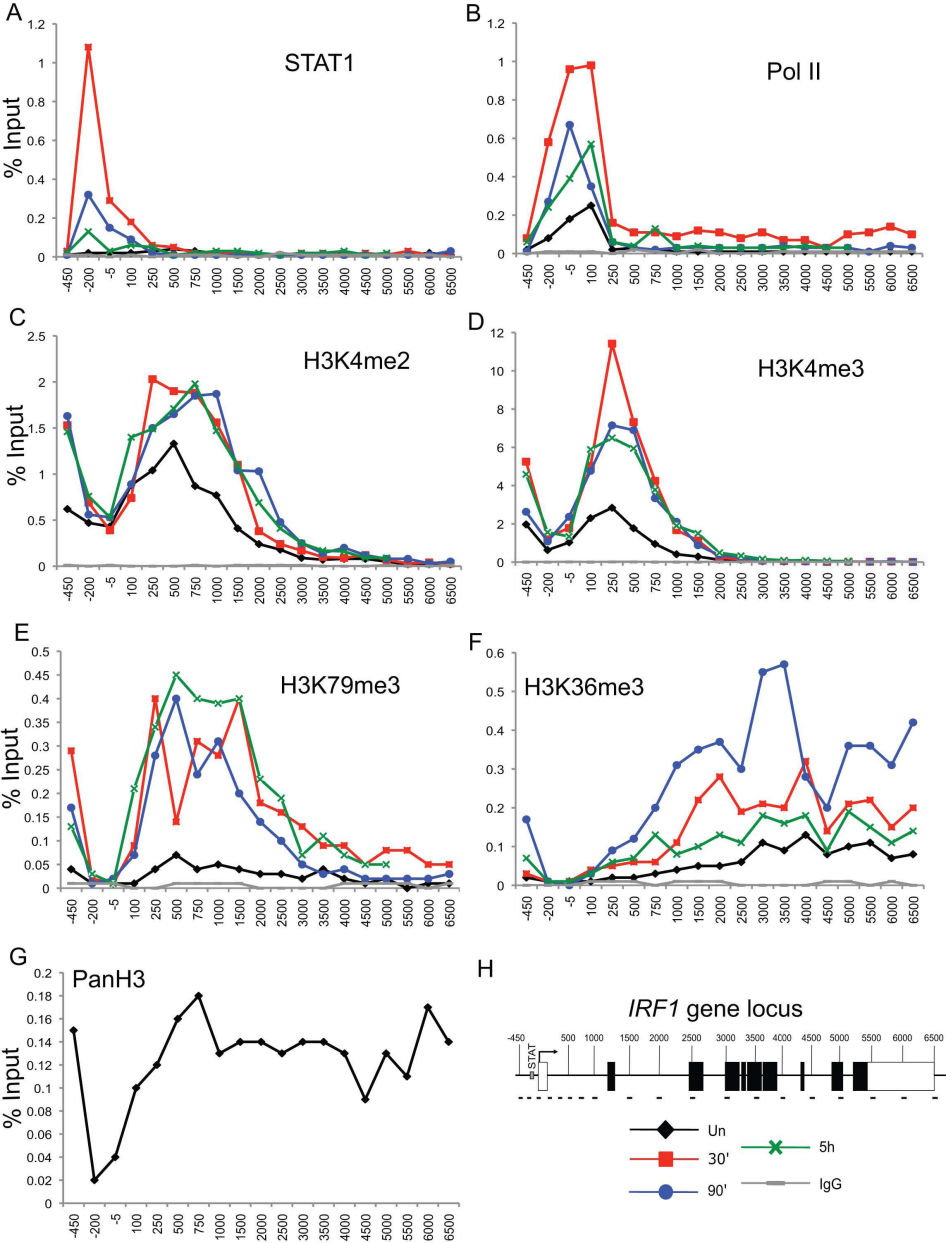
**Histone H3 lysine methylation across the interferon regulatory factor 1 gene (*IRF1*) locus is dynamic in response to interferon (IFN)γ induction**. **(a-g) **Chromatin immunoprecipitation (ChIP) of 2fTGH cells treated with IFNγ for 30 min, or uninduced, and then collected at various time points. The indicated antibodies were used and quantitative PCR (Q-PCR) quantified the precipitate yield, reported as percentage of input. IgG served as the negative control and Pan H3 antibody as the positive control and indicator of histone levels across *IRF1 ***(g)**. Locations of Q-PCR primers designed to span the entire locus of *IRF1 *are shown in **(h)**.

**Figure 3:**
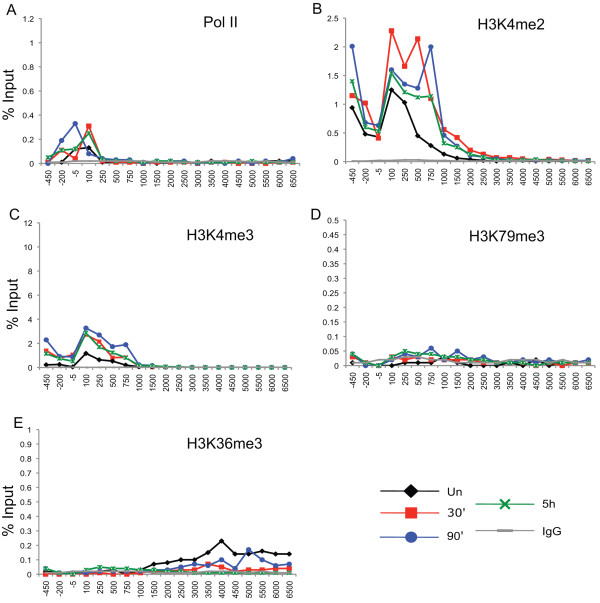
**Histone H3 lysine methylation is present across the interferon regulatory factor 1 gene (*IRF1*) locus but is not dynamic in signal transducer and activator of transcription 1 (STAT1)-null cells**. **(a-e) **Chromatin immunoprecipitation (ChIP) of U3A cells treated with interferon (IFN)γ for 30 min, or uninduced, and then collected at various time points. The indicated antibodies were used and quantitative PCR (Q-PCR) quantified the precipitate yield, reported as percentage of input. IgG served as the negative control. Locations of Q-PCR primers designed to span the entire locus of *IRF1 *are shown as in Figure 2h.

Studies from several model systems have determined that methylation of lysines 4, 36 and 79 of histone H3 are typically associated with active gene expression [9,11,18,34]. Likewise, we found H3K4me2, H3K4me3, H3K79me3 and H3K36me3 all increase in a manner that parallels STAT1 activity (Figure 2). With the exception of H3K79me3, the location of these modifications at the *IRF1 *locus is the same as reported in other studies [9,23]. H3K4me3 is located near the promoter, as is H3K4me2, although the latter shows the considerably broader peak typical for this modification (Figure 2c, d). H3K4me2 and me3 are also present at significant levels near the promoter in the uninduced state and in U3A cells, as is RNA polymerase II (Pol II) (Figures 2 and 3), confirming that *IRF1*, like most genes, undergoes transcriptional initiation [8,23]. The sharp dip observed at position -5 reflects the nucleosome depletion found at transcription start sites (TSSs).

H3K79me3 overlaps with H3K4me2 but extends further into the coding region, consistent with its described association with transcriptional elongation (Figure 2e) [19]. H3K79me3 is found only at very low levels at the *IRF1 *gene in 2fTGH cells in the uninduced state or in U3A cells (Figure 3). Thus, this modification specifically correlates with STAT1-induced transcription. This profile for H3K79me3, is different from the profile reported in a genome-wide study of CD4+ T cells which found H3K79me3 higher at silent promoters than at active ones, except in a narrow region near the TSS [9].

As expected, H3K36me3 was found to be biased toward the 3' region of the gene and is associated with elongation (Figure 2f). The H3K36me3 profile differs from the other profiles collected; specifically, this modification returns to uninduced levels at 5 h post-IFNγ treatment suggesting that a demethylase may target H3K36. The overall histone occupancy observed, determined using a pan H3 antibody, was consistent across the *IRF1 *locus, except at the TSS where a dip was observed as a result of TSS histone depletion (Figure 2g).

To confirm the dynamic nature of histone modification in STAT induced transcription, we performed ChIP assays at three other IFNγ responsive genes (*RING4*, *PSMB8*, *GBP1*) with quantitative real-time PCR (Q-PCR) primers designed to amplify the 5', 3' and middle regions of these gene loci and found similar patterns (data not shown). Taken together, the patterns and dynamics of these histone modifications indicate that STAT1 dependent genes experience transcription initiation but that downstream events, dependent upon STAT1's activation and DNA binding, regulate the accumulation of transcripts.

### Inhibition of methyltransferase activity decreases H3K4me3 and H3K36me3 at the *IRF1 *gene as well as *IRF1 *transcription

**Figure 4:**
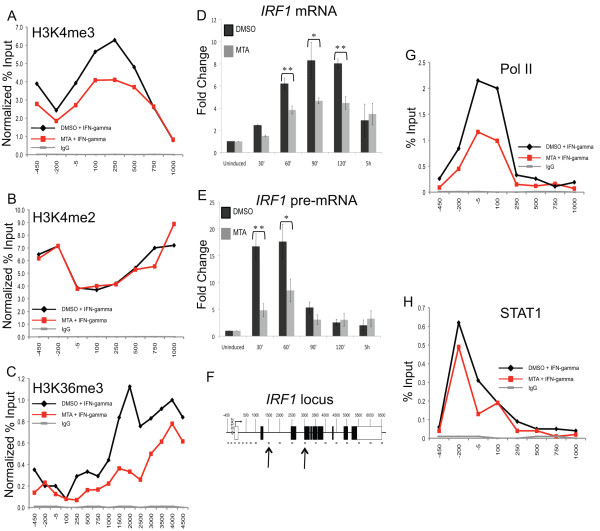
**Dynamic H3 lysine methylation at the activated interferon regulatory factor 1 gene (*IRF1*) and *IRF1 *transcription are altered by 5'-deoxy-5'-methyl-thioadenosine (MTA) treatment**. 2fTGH cells treated with MTA (1.5 mM) or dimethylsulfoxide (DMSO) for 24 h followed by treatment with interferon (IFN)γ for 30 min. **(a-c) **Chromatin immunoprecipitation (ChIP) using the indicated antibodies. Data were normalized to Pan H3 cycle threshold (Ct) values to account for a non-specific carrier (DMSO) effect and reported as percentage of input. **(d, e) **Reverse transcription quantitative PCR (RT Q-PCR) was performed after the times shown to quantitate *IRF1 *mRNA and pre-mRNA expression relative to glyceraldehyde 3-phosphate dehydrogenase gene (*GAPDH*) expression and presented as fold change upon induction. Error bars represent standard error (*n *= 3). **P = 0.01, *P = 0.05. **(f) **Arrows indicate locations of Q-PCR primers designed to amplify either exonic (d) or intronic **(e) **regions of the *IRF1 *gene. **(g, h) **RNA polymerase II (Pol II) and signal transducer and activator of transcription 1 (STAT1) ChIP of 2fTGH cells.

Next, we investigated whether the pharmacological drug, 5'-deoxy-5'-methyl-thioadenosine (MTA), which inhibits protein and DNA methylation, altered the dynamic changes in histone methylation observed at the *IRF1 *gene in response to IFNγ [35,36] Previously, MTA treatment had been shown to reduce the global levels of H3K4me3 by approximately twofold in HeLa cells, and to specifically reduce H3K4me3 but not H3K4me2 levels in the genome of HSV-1 during lytic infection [36]. Similarly, we observed that induced H3K4me3 levels were reduced by treatment with MTA, and that H3K4me2 remained unchanged (Figure 4a, b). However, the global levels of H3 lysine 4 methylation were not detectably lowered suggesting that the turnover of these modifications in 2fTGH cells is slower than in HeLa cells (Additional file 1a, b). We also examined the effect of MTA treatment on inducible H3K36me3 levels and found that they were decreased as well (Figure 4c). This is not surprising, given that MTA is a general methyltransferase inhibitor. (The ChIP-grade antibody used to immunoprecipitate H3K79me3 in Figure 2 was discontinued and so we were unable to follow up our studies of this modification.)

MTA has also been shown to decrease HSV-1 gene transcription during lytic infection [36]. Therefore, we considered if the decreases we observed for H3K4me3 and H3K36me3 correlated with decreased transcription of the *IRF1 *gene using reverse transcriptase followed by Q-PCR. Indeed, an approximately twofold reduction in the steady-state level of *IRF1 *mRNA was observed (Figure 4d). We confirmed that the reduction was due to decreased transcription by quantitating the *IRF1 *transcript levels in heterogeneous nuclear RNA (hnRNA) using PCR primers that target intronic sequences (Figure 4e) [37]. STAT1 occupancy was not altered by MTA treatment, but Pol II occupancy at the TSS was decreased (Figure 4g, h).

### H2B monoubiquitination

**Figure 5:**
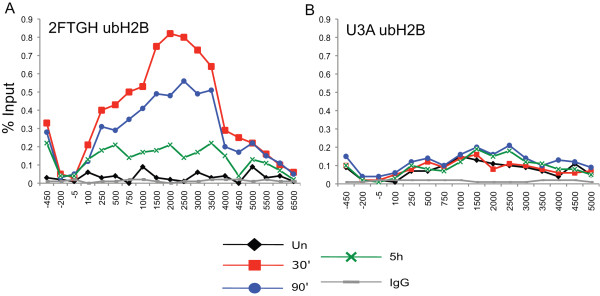
**H2B monoubiquitination is dynamic during interferon regulatory factor 1 gene (*IRF1*) induction**. Chromatin immunoprecipitation (ChIP), using ubH2B antibody, of 2fTGH cells **(a) **or U3A cells **(b) **treated with interferon (IFN)γ for 30 min and collected after the various time points. IgG served as the negative control. Locations of quantitative PCR (Q-PCR) primers designed to span the entire locus of *IRF1 *are as in Figure 2h.

In yeast, H3 K4 methylation depends upon H2B monoubiquitination [38]. In order to investigate the role of H2B ubiquitination in STAT1-induced transcription, we first determined the profile of ubH2B at the *IRF1 *locus at different times of IFNγ induction in 2fTGH cells (Figure 5a) and in U3A cells (Figure 5b). ubH2B quickly increases in response to IFNγ, but decreases at later time points. ubH2B is found highest in the middle coding region and lowest in the 5' and 3' regions. This profile is identical to the ubH2B profile described in other recent reports of mammalian cells, and the transient nature is similar to an observation that has been reported in the open reading frame (ORF) of the yeast *GAL1 *gene [26,27,39]. In U3A cells, ubH2B is also associated with the middle region of the gene, though at very low levels (Figure 5b). In fact, we recovered all assayed modifications (except perhaps H3K79me3) in the uninduced state and in the STAT1-null cell line (Figures 2 and 3). Thus, maintenance of these histone modifications does not depend upon the induction of IFNγ responsive genes. Their maintenance could be related to either basal transcription or a transcription-independent mechanism or some combination of both. Regardless, the observation of these modifications in uninduced cells and U3A cells agrees with other reports, and likely reflects how inducible genes are maintained in a repressed but poised state [8,23].

### RNF20 and a MLL/Menin complex are localized to *IRF1*

**Figure 6:**
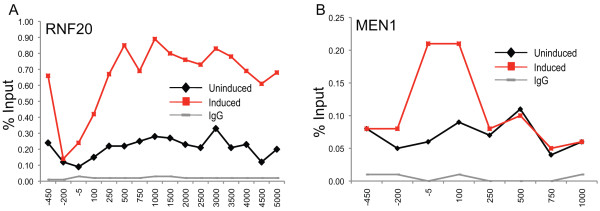
**RNF20 and MEN1 are constitutively associated with the interferon regulatory factor 1 gene (*IRF1*) and recruited by interferon (IFN)γ induction**. 2fTGH cells were induced with IFNγ for 30 min or uninduced. Chromatin immunoprecipitation (ChIP) was performed using antibodies against RNF20 **(a) **and MEN1 **(b) **and quantitative PCR (Q-PCR) quantified the precipitate yield, reported as percentage of input. MEN1 was not detectable using primer pairs beyond 3,000 bp.

Having observed that both H2B monoubiquitination and H3K4 methylation are dynamic during *IRF1 *gene expression, we next asked if the enzymes driving these histone modifications are associated with or recruited to the *IRF1 *gene as a result of IFNγ induction. A ChIP-grade antibody was used to immunoprecipitate RNF20 from chromatin collected from cells in both the uninduced and induced state (Figure 6a). In response to IFNγ, RNF20 accumulated across the *IRF1 *gene locus, beginning at around +250 bp, which is where ubH2B also begins to accumulate. Low levels of RNF20 were localized across the *IRF1 *gene in the uninduced state as well, suggesting that it might play a role in basal transcription.

There are several recognized H3K4 methyltransferases in mammalian systems, including SET1A/B and MLL1-4 [40]. However, ChIP-grade antibodies that discriminate among all of them are not available. Because the non-enzymatic proteins that contribute to COMPASS and COMPASS-like complexes do show some specificity, we used a ChIP-grade antibody against Menin (MEN1), which is a component unique to the MLL1 and MLL2 COMPASS-like complexes. ChIP assays showed that MEN1 is constitutively associated with the *IRF1 *promoter and that induction leads to an increase in a MEN1-containing complex (Figure 6b). The COMPASS-like activity constitutively associated with the *IRF1 *promoter, potentially explains the significant amounts of H3K4 methylation observed in uninduced cells and even in U3A cells (Figures 2 and 3).

### RNAi-mediated knockdown of RNF20 upregulates *IRF1 *transcription

**Figure 7:**
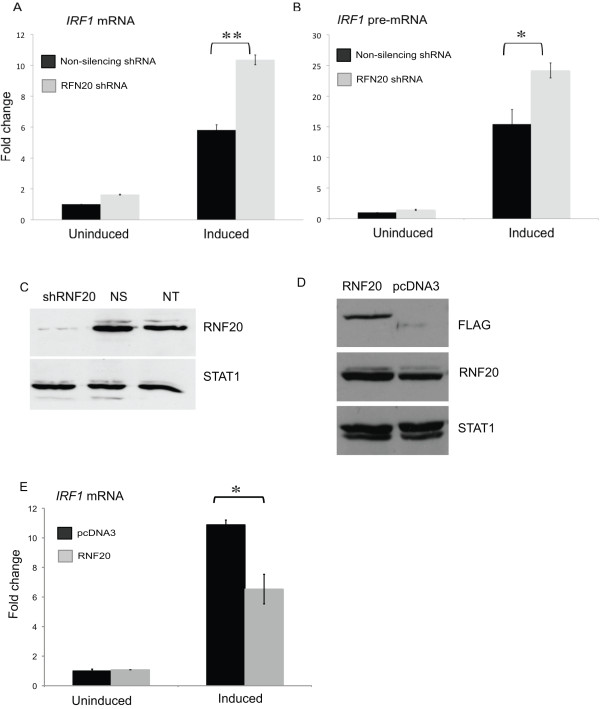
**RNAi-mediated RNF20 knockdown increases interferon regulatory factor 1 gene (*IRF1*) transcription**. **(a, b) **Reverse transcription quantitative PCR (RT Q-PCR) to quantitate *IRF1 *mRNA and pre-mRNA expression in stably selected 2fTGH cells expressing a non-silencing small hairpin (sh)RNA or *RNF20-shRNA *that were uninduced or treated with IFNγ. *IRF1 *expression was normalized to *ACTB *and presented as fold change relative to the uninduced, non-silencing shRNA condition. Error bars are standard error (*n *= 4). **(c) **Western blot of extracts prepared from stably selected 2fTGH cells expressing non-silencing shRNA (NS) or *RNF20 *shRNA or non-transfected cells (NT) with α-RNF20. Signal transducer and activator of transcription 1 (STAT1) served as loading control. **(d) **Western blot of extracts prepared from transiently transfected 2fTGH cells expressing pcDNA3.0 or FLAG tagged RNF20 pcDNA3.0 with α-FLAG and α-RNF20. STAT1 served as loading control. **(e) **RT Q-PCR of transiently transfected 2fTGH cells expressing pcDNA3.0 or FLAG tagged *RNF20 *pcDNA3.0 as in **(a)**. Error bars are standard error (*n *= 2). **P = 0.01, *P = 0.05. Blot quantifications were performed using ImageJ.

Having observed that RNF20 was recruited to *IRF1 *by IFNγ induction, we initially hypothesized that RNAi-mediated knockdown of RNF20 would lower or prevent *IRF1 *expression. Stable transfection of 2fTGH cells with pGIPZ small hairpin RNA (*shRNA*)*-RNF20 *reduced the endogenous expression of RNF20 by 98% (Figure 7c). However, we consistently observed increased inducible transcription in *shRNA-RNF20 *cells compared to a cell line stably transfected with a non-silencing construct (Figure 7a). We confirmed that the increased *IRF1 *mRNA accumulation was due to increased transcription using intronic primers (Figure 7b). When an *RNF20 *cDNA that is C-terminally FLAG tagged was transiently overexpressed (40%, Figure 7d) in 2fTGH cells, the opposite effect on activated *IRF1 *transcription was observed (Figure 7e).

### H2B monoubiquitination and H3 lysine methylation

**Figure 8:**
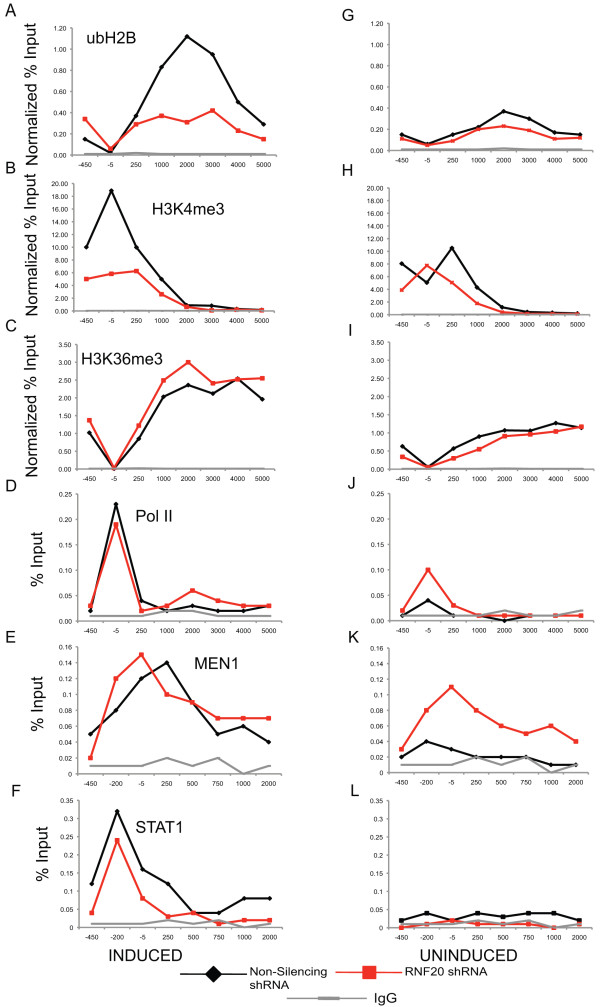
**RNAi-mediated *RNF20 *knockdown prevents inducible ubH2B and H3K4me3**. **(a-l) **Chromatin immunoprecipitation (ChIP) using the indicated antibodies of stably selected 2fTGH cells expressing a non-silencing shRNA or *shRNA-RNF20 *after induction with interferon (IFN)γ or uninduced. Except for MEN1 **(k)**, there were no statistically significant differences in any of the uninduced ChIP assays.

RNF20 knockdown diminished ubH2B during induction of the *IRF1 *gene (Figure 8a). Importantly, inducible H3K4me3 was lost as well (Figure 8b), providing support for a crosstalk mechanism in which H2B monoubiquitination promotes H3K4 methylation, as has been described in other systems previously [38]. RNF20 knockdown did not affect H2K4me2 in any condition (data not shown) and the uninduced levels of ubH2B, H3K4me3 were also unchanged in the *shRNA-RNF20 *cell line compared to the non-silencing control cell line (Figure 8g, h). H3K36me3 showed the same profiles in both the induced and uninduced conditions (Figure 8c, i). Pol II, MEN1 and STAT1 are recruited properly in response to IFNγ in these cell lines (Figure 8d-f), although MEN1 does appear to acquire an increased stability or association in the uninduced state in the *shRNA-RNF20 *cell line (Figure 8k), but this enhanced association does not correlate with increased H3K4me3 (Figure 8h). The RNF20 knockdown achieved in this cell line is insufficient to fully ablate ubH2B; western blotting demonstrated that there was an approximately 65% loss of global ubH2B in the *shRNA-RNF20 *cell line (Additional file 1c). An alternative explanation is that hBRE1 is not the only H2B ubiquitinase in 2fTGH cells, although this is unlikely [26].

Supportive of a crosstalk mechanism where ubH2B promotes H3K4 methylation, we note that inhibition of H2B ubiquitination using carbobenzoxy-L-leucyl-L-leucyl-L-leucinal (MG132), a proteosome inhibitor that has been used to rapidly and efficiently deplete ubH2B [27,41], correlated with loss of both H3K4me3 and H3K4me2 (Additional file 2b, c) in response to IFNγ, while H3K36me3 was unaffected (Additional file 2d). When 2fTGH cells were treated with MG132, ubH2B was eliminated globally (Additional file 1d), and at the *IRF1 *gene locus when cells were induced with IFNγ (Additional file 2a).

Genetic studies in yeast and mammalian *in vitro *transcription assays have demonstrated that H2B monoubiquitination depends upon the early steps of transcriptional elongation, requiring the presence of the polymerase associated factor (PAF) complex and the addition of nucleoside triphosphates (NTPs), and not simply recruitment of Rad6/Bre1 [26,38]. To find out if ubH2B at the *IRF1 *gene also depends upon elongation, 2fTGH cells were treated with the elongation inhibitor 5,6-dichlorobenzimidazole riboside (DRB) [16,23]. Pol II promoter occupancy is unaffected by DRB treatment [18]. We confirmed that DRB ablates *IRF1 *induced gene expression. (Additional file 3a) and it also significantly inhibits basal transcription. In ChIP assays, in the DRB treated condition, induced ubH2B was strongly decreased, as was H3K4me3 (Additional file 3b, c). H3K36me3 normally correlates with ongoing transcription and so, not surprisingly, induced H3K36me3 was also decreased by DRB (Additional file 3d).

### RNAi-mediated knockdown of RNF20 alters Pol II C-terminal domain (CTD) phosphorylation

In yeast, H2B ubiquitination has been functionally tied to transcriptional elongation; H2B ubiquitination/deubiquitination occurs dynamically, with deubiquitination required for the recruitment of the RNA polymerase II CTD serine 2 kinase, Ctk1[39,42]. Additionally, H3K4 methylation has been attributed a repressive role at the *GAL10-GAL1 *locus [43]. H3K4me2/3 occurs via cryptic transcription and recruits a histone deacetylase (HDAC) activity (Rpd3) to dampen *GAL1 *promoter activity by inhibiting Pol II recruitment. In the absence of H3K4 methylation, *GAL1 *induction is accelerated.

**Figure 9:**
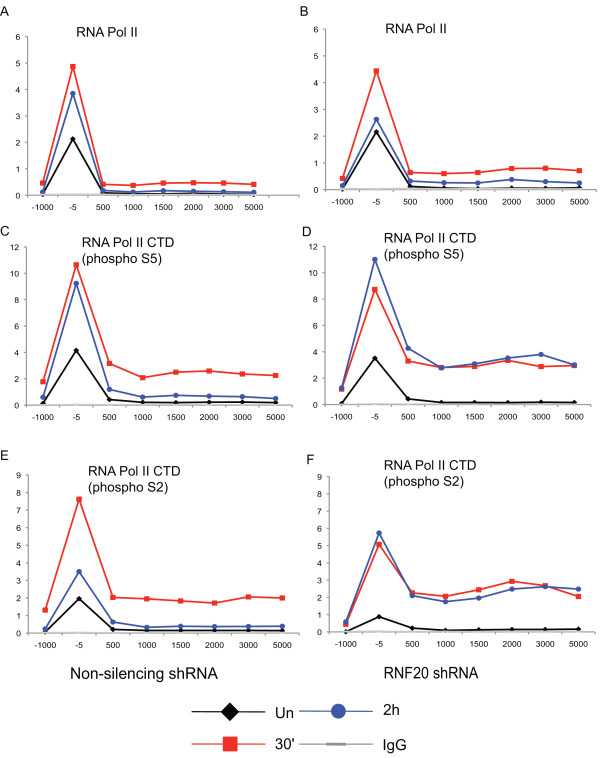
**RNA polymerase II (Pol II) C-terminal domain (CTD) serine phosphorylation in signal transducer and activator of transcription 1 (STAT1)-activated interferon regulatory factor 1 gene (*IRF1*) transcription is altered by RNF20 knockdown**. Chromatin immunoprecipitation (ChIP) of stably selected 2fTGH cells expressing a non-silencing small hairpin (shRNA) or *shRNA-RNF20 *after induction with interferon (IFN)γ or uninduced using antibodies specific for total Pol II (a, b), the serine 5 phosphorylated CTD of Pol II (c, d) and the serine 2 phosphorylated CTD of Pol II (e, f). The same results are seen when the data are normalized to total Pol II.

Because H2B monoubiquitination is transient and peaks prior to maximal *IRF1 *transcription (Figure 5a), which occurs at approximately 90 min, and RNF20 depletion upregulates *IRF1 *while decreasing H3K4me3, we speculated that RNF20 might directly or indirectly, affect the recruitment of Pol II and/or the dynamics of the phosphorylation that occurs at the CTD of Pol II during transcription. As Pol II moves across a locus, a change in phosphorylation occurs in the repeated sequence, YSPTSPS, in the CTD of Pol II; serine 5 is phosphorylated at initiation, while serine 2 phosphorylation is added during elongation [44,45]. ChIP assays using antibodies that recognize total Pol II, serine 2 phosphorylation and serine 5 phosphorylation in the CTD were performed using chromatin harvested at different times of *IRF1 *gene activation (Figure 9). The total Pol II levels change as expected, increasing early in gene induction and decreasing as transcription wanes at the later time point, but with no differences in the *shRNA-RNF20 *cell line versus the non-silencing cell line (Figure 9a, b). Phosphoserine 5 Pol II and phosphoserine 2 Pol II also behave identically in the two cell lines at 30 min of IFNγ induction and in the uninduced condition (Figure 9c-f). However, at the later time point, both phosphoserine 5 Pol II and phosphoserine 2 Pol II in the RNF20 depleted cell line remain high and do not return to the lower levels seen in the control cell line (Figure 9d, f). Based on these data, we suspect that the increase in transcription in the RNF20 depleted cells is mainly the result of loss of a repressive effect from ubH2B. Were the observed increase in transcription due to an H3K4me2/3 based mechanism, Pol II and phosphoserine 5 Pol II should have been enriched at 30 min and/or in the basal state. One possibility, among many, is that ubH2B, in addition to determining the accessibility of a serine 2 kinase (positive transcription elongation factor b (P-TEFb)) to Pol II to foster elongation, also determines the accessibility of the phosphatases that target serine 2 and/or 5. The result is a defect in Pol II CTD cycling that acts to promote ongoing transcription.

## Discussion

In this study, we characterized several histone modifications in STAT1 activation of the *IRF1 *gene and showed that ubH2B promotes H3K4me3 during induction. Additionally, the enzymatic activities that produce these modifications, COMPASS-like and RNF20, are found at the *IRF1 *gene and are further recruited by induction. These data, along with the observation that methyltransferase inhibition hinders *IRF1 *transcription, suggested a positive regulatory role for these enzymes. Unexpectedly, the E3 ligase that targets H2B for ubiquitination, RNF20, acts as a repressor of transcription, ostensibly by altering the dynamic phosphorylation of the CTD of Pol II that determines its initiation, elongation and termination.

### Mammalian ubH2B function

Other studies of ubH2B in inducible mammalian gene expression have suggested alternative roles for this modification, than the repressive role our data indicates. Using *in vitro *transcription assays with a recombinant, fully H2B-ubiquitinated chromatin template, Kim *et al*. observed that H2B ubiquitination does not affect p53 activated transcription, arguing that H2B ubiquitination is a consequence of transcription and it does not directly affect the function of the transcriptional machinery [26]. In contrast, ubH2B reportedly enhances the rate of transcriptional elongation by Pol II in response to retinoic acid [46], and at *HOX *genes [47]. The dynamics of H2B ubiquitination during transcriptional activation (Figure 5) and the observation that RNF20 knockdown increases inducible *IRF1 *transcription (Figure 7a, b), while its overexpression inhibits *IRF1 *(Figure 7e) all argue against the ideas that this modification occurs in the wake of Pol II or promotes Pol II passage at the *IRF1 *gene. It is possible that these disparities are simply related to the use of *in vitro *assays that differently or incompletely recapitulate the inducible transcription that occurs *in vivo*. Alternatively, the function of ubH2B in gene expression might be context dependent.

### RNF20 as repressor in mammalian gene expression

Using gene expression microarrays, Shema *et al*. identified two cohorts of genes that are selectively up or downregulated by RNF20 depletion in HeLa cells [28]. Based on our results, we speculate that these two cohorts of genes may have different barriers built into their chromatin, which contribute to regulation of their expression. For instance, the cohort of genes that is activated by RNF20, such as *TP53 *and the *HOX *genes [28,47] may not require a repressive mechanism to be generated by ubH2B to regulate their transcription and, in those cases, ubH2B works to promote elongation. However, genes normally suppressed by RNF20 are likely to be poised for activation and undergo basal transcription, and therefore rely on H2B monoubiquitination as a check against inappropriately activated transcription. It is worth noting that Shema *et al*. report that this later cohort of genes is populated by several early response and proliferative genes, is enriched in Pol II and H3K4me3 (as is *IRF1*) as compared to RNF20 dependent and independent genes, and that many are constitutively 'on' but in a 'low gear' state, reflecting the unique features of these genes that poise them for rapid activation in response to signaling events.

### Similarities to activated transcription at the *GAL1 *gene in *Saccharomyces cerevisiae*

The role of histone modification in inducible gene expression is best characterized at the *GAL1 *locus in *Saccharomyces cerevisiae *[38]. At that gene, dimethylation and trimethylation of histone H3K4 depend upon monoubiquitination of H2B (ubH2B) at K123 in a mechanism involving the Rad6/Bre1 ubiquitination enzymes and the Set1/COMPASS methyltransferase complex [40]. While the precise mechanism for this trans-tail communication is not fully established, a model has emerged whereby the H2B ubiquitin ligase, Bre1, and the E2 ubiquitin conjugase, Rad6, are recruited to promoters by interaction with activators, such as Gal4 [38]. The enzymatic activity for the monoubiquitination of H2B, however, depends on further interactions with the PAF complex, the Bur complex and the phosphorylation of the CTD of Pol II at serine 5. H2B ubiquitination then recruits Cps35, (aka Swd2) an essential component of COMPASS, which leads to H3K4 dimethylation and trimethylation by the methyltransferase, Set1 [42,48-51]. H3K4me3 recruits the SAGA complex, which contains the deubiquitinase, Ubp8. Deubiquitination of ubH2B allows Ctk1 to phosphorylate the CTD of Pol II at serine 2, which in turn recruits the methyltransferase for H3K36, Set2 [39]. Thus, Ubp8 provides the opposing deubiquitinase activity that is required for transition to efficient transcriptional elongation at the inducible *GAL1 *gene. ubH2B's transitory profile at the *IRF1 *gene indicates that it might be removed by an opposing ubiquitin hydrolase, as it is in yeast. While other events, such as histone exchange or direct or indirect RNF20 inactivation, are also possible, studies in the lab are ongoing to determine if USP22 (the mammalian homolog to Ubp8) also deubiquitinates ubH2B. It seems a likely possibility it does, since USP22 is required for MYC activated transcription [52], but whether ubH2B directly blocks recruitment of a kinase (P-TEFb) responsible for serine 2 phosphorylation in CTD of Pol II is not known [53].

Several other important research questions are suggested by the data presented here. First, what is the mechanistic basis for the defect observed in Pol II CTD phosphorylation cycling and is this the direct cause of increased transcription observed in the RNF20 depleted cell line? Might H2B monoubiquitination also impact the function of CTD phosphatases (Fcp1 and Rtr1) as it does the kinase (Ctk1) across gene loci [54]? Second, what is the basis for RFN20's recruitment? Does STAT1 help to recruit hBRE1, as Gal4 and p53 do [38,55]? What is the role of the PAF complex? And what is the precise nature of the MEN1-containing, COMPASS-like complex and which of its components functions as the translator in the crosstalk with hRAD6/hBRE1? WDR82 mediates the crosstalk between hSET1/COMPASS and the ubiquitination complex, but it is a component specific to hSET1 [25]. While we cannot rule out the possibility that SET1/COMPASS, or another H3K4 methyltransferase, is also involved redundantly, the colocalization of MEN1 and RNF20 at the *IRF1 *gene is the first evidence of a possible interaction between COMPASS-like and hBRE1 in mammalian gene expression.

### A different role for H3K36me3

Of the several histone modifications we profiled in this study, only ubH2B and H3K36me3 return to basal levels, mirroring the dynamics of STAT1 activation (Figures 2a, f and 5a) and *IRF1 *transcription (Figure 1). In yeast, H2B deubiquitination drives H3K36me2, which then works to prevent cryptic transcription by Pol II in the 3' end of genes by recruiting a histone deacetylase [56-58]. In addition, H3K36me2 levels do not parallel transcriptional output in a titratable fashion, but rather reflect one of two states: uninduced/basal transcription or induced transcription [59,60]. We, and Edmonds *et al*. [23], observed this same profile for H3K36me3 at inducible genes in mammalian cell lines. However, mammalian H3K36me1/me2 levels are very low, and deletion of the Set2 homolog responsible for H3K36me3, SETD2, removes all H3K36me3, but with no consequence for proper transcription, leading Edmunds *et al*. to conclude that K36 methylation in mammalian transcription does not mirror its role in yeast. Our data support this conclusion, given that forced H2B deubiquitination, achieved with MG132 and RNF20 knockdown, did not alter H3K36me3.

While it is not yet clear what H3K36me3 contributes to mammalian inducible gene expression, recent research has revealed that H3K36me3 preferentially marks exons relative to introns and it has been proposed that H3K36me3 exon marking connects transcription and splicing [61,62]. While the primer pairs used to profile the *IRF1 *gene were designed without considering intron-exon structure, the exons are heavily biased to the 3' end in the *IRF1 *gene (Figure 2h). The 3' bias observed for H3K36me3 may reflect this.

## Conclusions

Signal transduction pathways, like the JAK-STAT pathway, relay signals from the extracellular environment through the cytoplasm and ultimately to the DNA, which is organized as chromatin in the nucleus. Chromatin then serves as the template for dynamic nuclear signaling events (that is, post-translational modifications of histones) that regulate transcription. These dynamic signaling events are highly integrated, and it is becoming more clear that it is the proper balance between opposing enzymatic activities (E3 ubiquitin ligase/ubiquitin hydrolase, HMT/HDM, kinase/phosphatase and so on) that determine the functional output of a histone modification as either activating or repressing [63]. Therefore, it is important to better define the function of histone modifications and the interplay among the enzymatic activities (COMPASS-like, hBRE1, PAF1 complex, USP22, and so on) that promote these modifications if we are to fully understand how chromatin contributes to both normal and aberrant activated transcription in mammalian systems.

## Methods

### Antibodies

The antibodies used were as follows: H3K4me3 (Abcam, Cambridge, MA, USA, ab8580), H3K4me2 (Millipore, Billerica, MA, USA, 07-030), H3K36me3 (Abcam ab9050), H3K79me3 (Abcam ab2621), Pan H3 CT (Millipore 07-690), ubH2B (Medimabs, Quebec, Canada, MM-0029-p), RNA Pol II (Santa Cruz, Santa Cruz, CA, USA sc-899), RNA polymerase II CTD repeat YSPTSPS (phospho S2) (Abcam ab5095), RNA polymerase II CTD repeat YSPTSPS (phospho S5) (Abcam ab5131), IgG (Jackson Immunoresearch, West Grove, PA, USA), STAT1 (Santa Cruz sc-345X), phospho-STAT1 (Tyr701) (Cell Signaling Technology, Danvers, MA, USA, 9171S), Menin (Bethyl Laboratories, Montgomery, TX, USA, A300-105A), RNF20 (Abcam ab70495), RNF20-ChIP-grade (Novus Biologicals, Littleton, CO, USA, NB100-2242), FLAG (Sigma), Anti-rabbit or anti-mouse horseradish peroxidase (HRP) (Jackson Immunoresearch).

### Cell lines and chemical inhibitors

2fTGH and U3A cell lines were cultured in HyClone Dulbecco's modified Eagle medium (DMEM)/high glucose media supplemented with 10% cosmic calf serum and 10% antibiotic/antimycotic (Fisher Scientific, Pittsburgh, PA, USA). Interferon (IFN)γ treatment in all cases involved adding IFNγ (R&D Systems, Minneapolis, MN, USA, 5 ng/ml) to the media for 30 min, replacing with fresh media and harvesting cells at the indicated times. MTA (Sigma, St. Louis, MO, USA), MG132 (Calbiochem, San Diego, CA, USA), DRB (Sigma) treated cells were prepared as indicated in the figure legends.

### Reverse transcriptase Q-PCR

Total RNA was collected using Isol-RNA lysis reagent (5 Prime, Gaithersburg, MD, USA). RNA was DNaseI (Invitrogen, Carlsbad, CA, USA) treated and phenol/chloroform extracted. RNA (2 μg) was converted to cDNA using the High Capacity RNA-to-cDNA kit (Applied Biosystems, Carlsbad, CA, USA). cDNA was then subjected to Q-PCR (Sybr Green, 7500 FAST Real Time PCR System, Applied Biosystems) using gene specific primers to the intronic or exonic region of the *IRF1 *gene. In all cases, an RT(-) control confirmed no DNA contamination. Primer sequences will be provided upon request. PCR efficiency was determined for all primer pairs before their use. To ensure the statistical significance of differences reported in the RT-Q-PCR assays, standard errors were calculated for the multiplicates, and when SE bars did not overlap, a paired *t *test confirmed significance, *P *≤ 0.05.

### Western blot analysis

Cells were collected after various treatments and whole cell extract, prepared as described in [64], was subjected to SDS-PAGE (30 μg) and transferred to a nitrocellulose membrane. Immunodetection was performed using anti-pan H3 CT (1:25,000), anti-ubH2B (1:1,000), anti-H3K4me3 (1:500), anti-H3K4me2 (1:5,000), anti-RNF20 (1:5,000), anti-STAT1 (1:1,000), anti-pY-STAT1 (1:1,000). A HRP anti-species secondary antibody (1:10,000) was then applied and immunoreactive proteins were visualized using chemiluminescence reagent (Thermo Scientific, Pittsburgh, PA, USA). Histone acid extraction was carried out as described previously [65]. Bands were quantified with ImageJ.

### ChIP

ChIP was performed as described previously [66]. Briefly, 1 × 10^7 ^cells were fixed in 1% formaldehyde for 10 min followed by the addition of 0.125 M glycine. Cells were lysed using a douncer and the fixed chromatin was sheared by sonication. Chromatin was subject to centrifugation (13,000 rpm for 25 min at 4°C) and was then incubated overnight with various antibodies. Pan H3 and IgG were included in all ChIPs as positive and negative controls. Immunoprecipitation was carried out with protein A agarose/salmon sperm beads (Millipore). After washing, the chromatin was eluted from the beads and the cross links were reversed by heating at 65°C overnight. DNA was treated with RNase A and proteinase K (5 Prime), purified via phenol/chloroform extraction, precipitated with ethanol overnight and resuspended in TE buffer. Samples were analyzed by quantitative real time-PCR (Applied Biosystems) using gene specific primers designed to run the length of the *IRF1 *gene. Primer sequences provided upon request. PCR efficiency was determined for all primer pairs before their use. Data are expressed as percentage of input and all experiments were performed in duplicate, if not triplicate. Where indicated the data were normalized to the Pan H3 levels. To ensure the statistical significance of differences reported in the ChIP assays, standard errors were calculated for the multiplicates and, if necessary, a paired *t *test confirmed significance, *P *≤ 0.05.

### Transfection of shRNAmir and expression vectors

A pGIPZ shRNAmir vector targeting *RNF20 *mRNA (RHS4430-98486410) as well as a non-silencing shRNA vector (RHS4346) were purchased from Open Biosystems, Huntsville, AL, USA. C-terminally FLAG tagged RNF20 was PCR cloned between the *Kpn*I and *Eco*RV sites of pcDNA3.0 (Invitrogen) using an RNF20 cDNA as template (Open Biosystems, MHS4426-98361130). Transfection of 2fTGH cells was carried out using Arrest-in reagent according to the manufacturer's protocol (Open Biosystems). Puromycin was used to select for stable shRNAmir cell lines and individual clones were characterized according to their RNF20 protein expression using western blotting.

## Competing interests

The authors declare that they have no competing interests.

## Authors' contributions

LJB and EC carried out the experimental studies and helped to draft the manuscript. MAH conceived of the study, and participated in its design and coordination and wrote the manuscript. All authors read and approved the final manuscript.

## Supplementary Material

Additional file 1 **Western blotting of histones acid extracted after treatment with 5'-deoxy-5'-methyl-thioadenosine (MTA), carbobenzoxy-L-leucyl-L-leucyl-L-leucinal (MG132), and RNAi-mediated knockdown of RNF20 and of phosphorylated signal transducer and activator of transcription 1 (STAT1) during interferon (IFN)γ treatment**. **(a-d) **Acid-extracted histones collected from 2fTGH cells after various treatments were subjected to SDS-PAGE, blotted on nitrocellulose and the indicated antibodies were used to develop the blots. **(a, b) **MTA (1.5 mM for 24 h), dimethylsulfoxide (DMSO) (vehicle for MTA, for 24 h). **(c) **2fTGH cell lines stably expressing pGIPZ small hairpin RNA (*shRNA*)*-RNF20 *(RNF20), a non-silencing shRNA vector (NS) and cells not transfected (NT). *shRNA-RNF20*, 65% loss of total ubH2B. **(d) **MG132 (50 mM, for 4 h) produced a 95% loss of ubH2B compared to ethanol (vehicle for MG132, for 4 h). **(e) **Whole cell extracts of 2fTGH cells uninduced or induced with IFNγ for 30 min and returned to normal growth media were subjected to SDS-PAGE, blotted on nitrocellulose and a phosphotyrosine 701 STAT1 antibody was used to develop the blots. Upper band: STAT1α (91 kDa); lower band: STAT1β (84 kDa). Click here for file

Additional file 2 **Carbobenzoxy-L-leucyl-L-leucyl-L-leucinal (MG132) treatment decreases inducible H2B monoubiquitination, affecting H3K4 methylation but not H3K36 methylation**. **(a-d) **Chromatin immunoprecipitation (ChIP), using the indicated antibodies, of 2fTGH cells treated with MG132 (in ethanol, 50 mM) for 4 h or carrier and then induced with interferon (IFN)γ for 30 min. Click here for file

Additional file 3 **5,6-Dichlorobenzimidazole riboside (DRB) inhibition of transcription elongation prevents dynamic ubH2B, H3K4me3 and H3K36me3 during interferon regulatory factor 1 gene (*IRF1*) induction**. **(a) **2fTGH cells were treated with DRB (in ethanol, 25 mg/ml) for 10 min prior to induction with interferon (IFN)γ. Cells were collected and RT Q-PCR was performed to quantitate *IRF1 *expression relative to *GAPDH *and presented as fold change upon induction. Error bars represent standard error (*n *= 2). *P = 0.05. **(b-d) **2fTGH cells were treated with DRB for 10 min prior to induction with IFNγ. ChIP assays with ubH2B, H3K4me3 and H3K36me3 antibodies were performed and real-time quantitative PCR (Q-PCR) quantified the precipitate yield, reported as percentage of input. Click here for file
