# Supplementary figures and images for: Menin and RNF20 recruitment is associated with dynamic histone modifications that regulate signal transducer and activator of transcription 1 (STAT1)-activated transcription of the interferon regulatory factor 1 gene (IRF1)
Source: Epigenetics Chromatin. 2010 Sep 8;3:16. doi: 10.1186/1756-8935-3-16 (PMC2940767; doi:10.1186/1756-8935-3-16)

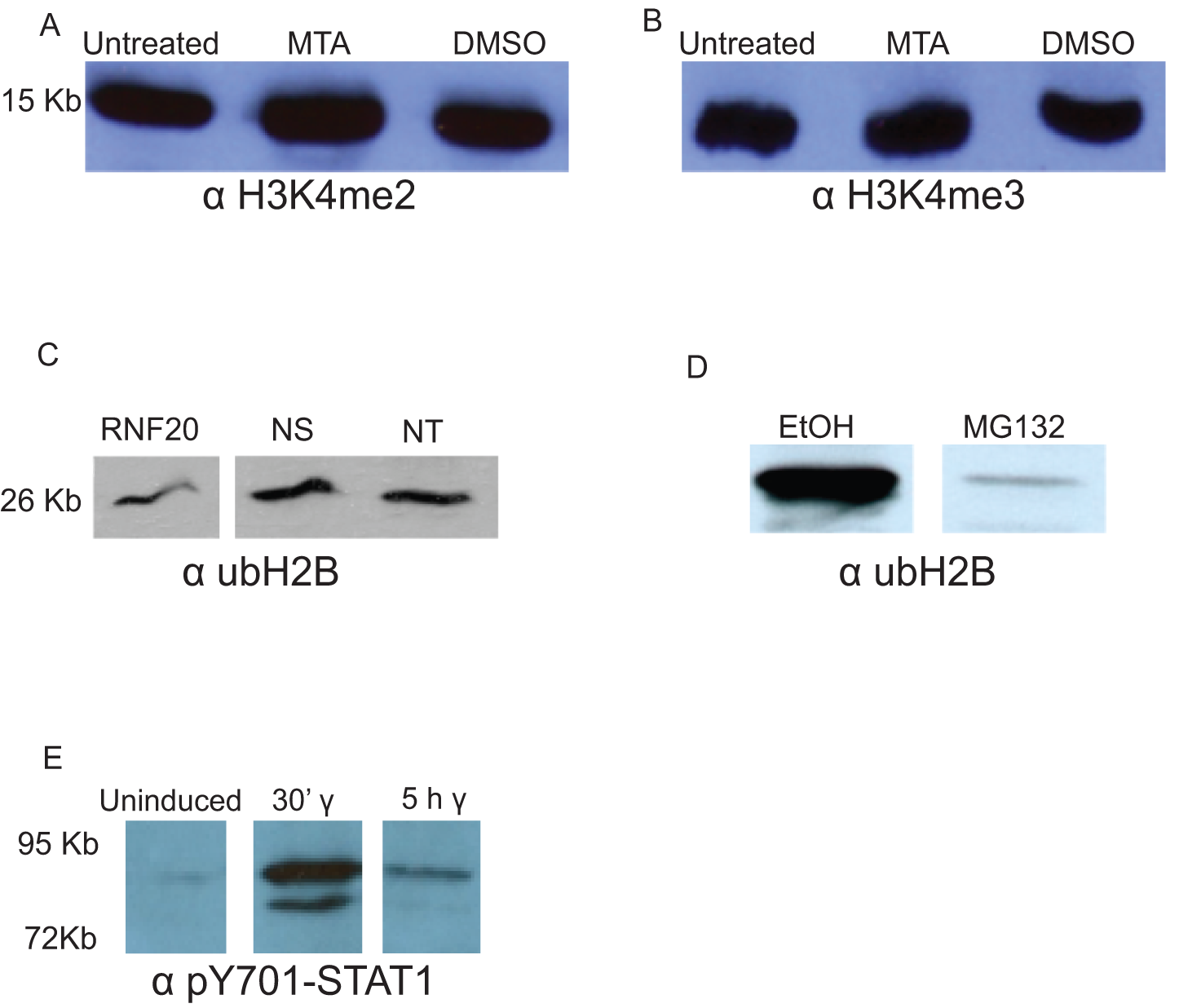

Supplement: Additional file 1 — Western blotting of histones acid extracted after treatment with 5'-deoxy-5'-methyl-thioadenosine (MTA), carbobenzoxy-L-leucyl-L-leucyl-L-leucinal (MG132), and RNAi-mediated knockdown of RNF20 and of phosphorylated signal transducer and activator of transcription 1 (STAT1) during interferon (IFN)γ treatment. (a-d) Acid-extracted histones collected from 2fTGH cells after various treatments were subjected to SDS-PAGE, blotted on nitrocellulose and the indicated antibodies were used to develop the blots. (a, b) MTA (1.5 mM for 24 h), dimethylsulfoxide (DMSO) (vehicle for MTA, for 24 h). (c) 2fTGH cell lines stably expressing pGIPZ small hairpin RNA (shRNA)-RNF20 (RNF20), a non-silencing shRNA vector (NS) and cells not transfected (NT). shRNA-RNF20, 65% loss of total ubH2B. (d) MG132 (50 mM, for 4 h) produced a 95% loss of ubH2B compared to ethanol (vehicle for MG132, for 4 h). (e) Whole cell extracts of 2fTGH cells uninduced or induced with IFNγ for 30 min and returned to normal growth media were subjected to SDS-PAGE, blotted on nitrocellulose and a phosphotyrosine 701 STAT1 antibody was used to develop the blots. Upper band: STAT1α (91 kDa); lower band: STAT1β (84 kDa). [file 1756-8935-3-16-S1.PNG]

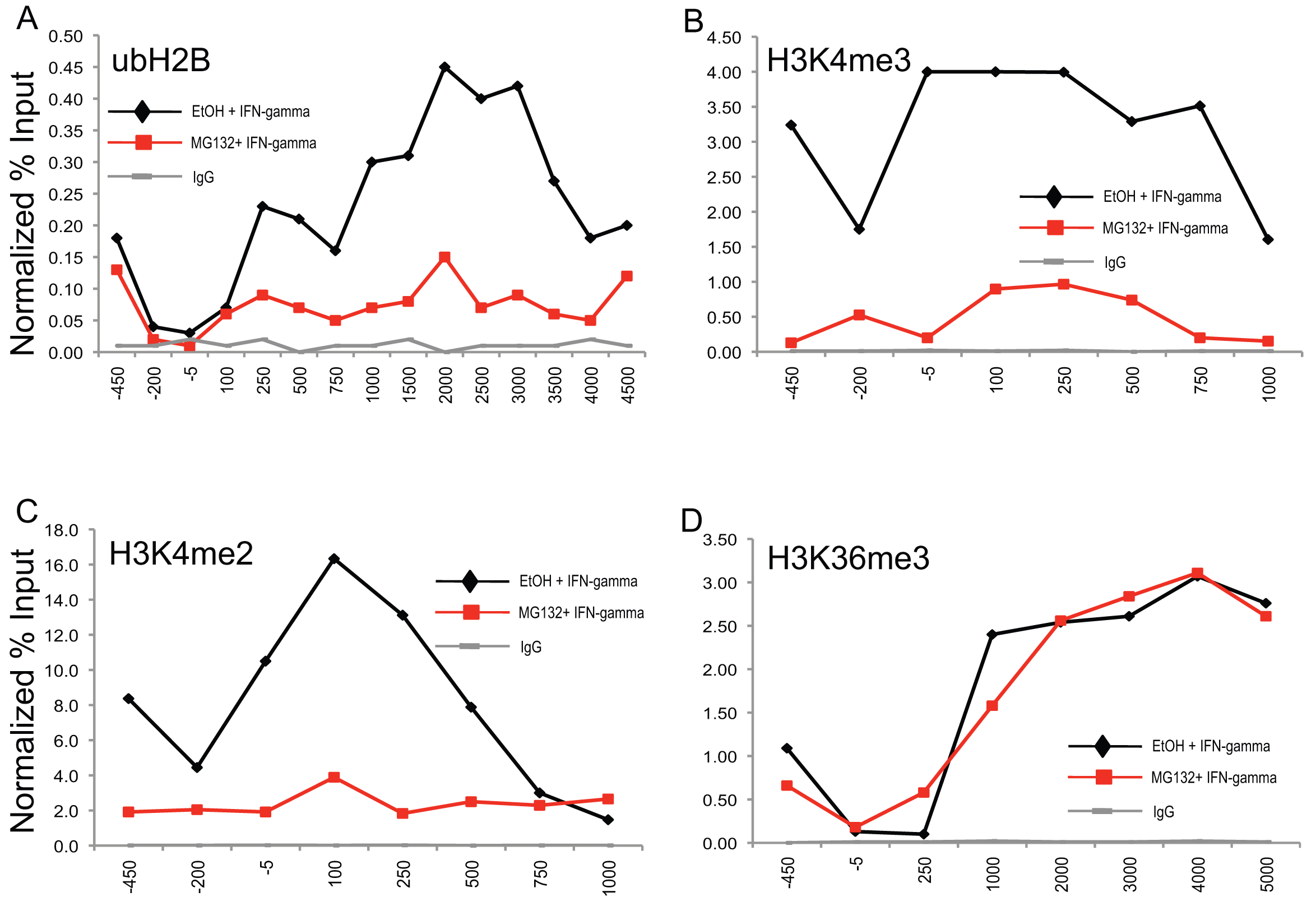

Supplement: Additional file 2 — Carbobenzoxy-L-leucyl-L-leucyl-L-leucinal (MG132) treatment decreases inducible H2B monoubiquitination, affecting H3K4 methylation but not H3K36 methylation. (a-d) Chromatin immunoprecipitation (ChIP), using the indicated antibodies, of 2fTGH cells treated with MG132 (in ethanol, 50 mM) for 4 h or carrier and then induced with interferon (IFN)γ for 30 min. [file 1756-8935-3-16-S2.PNG]

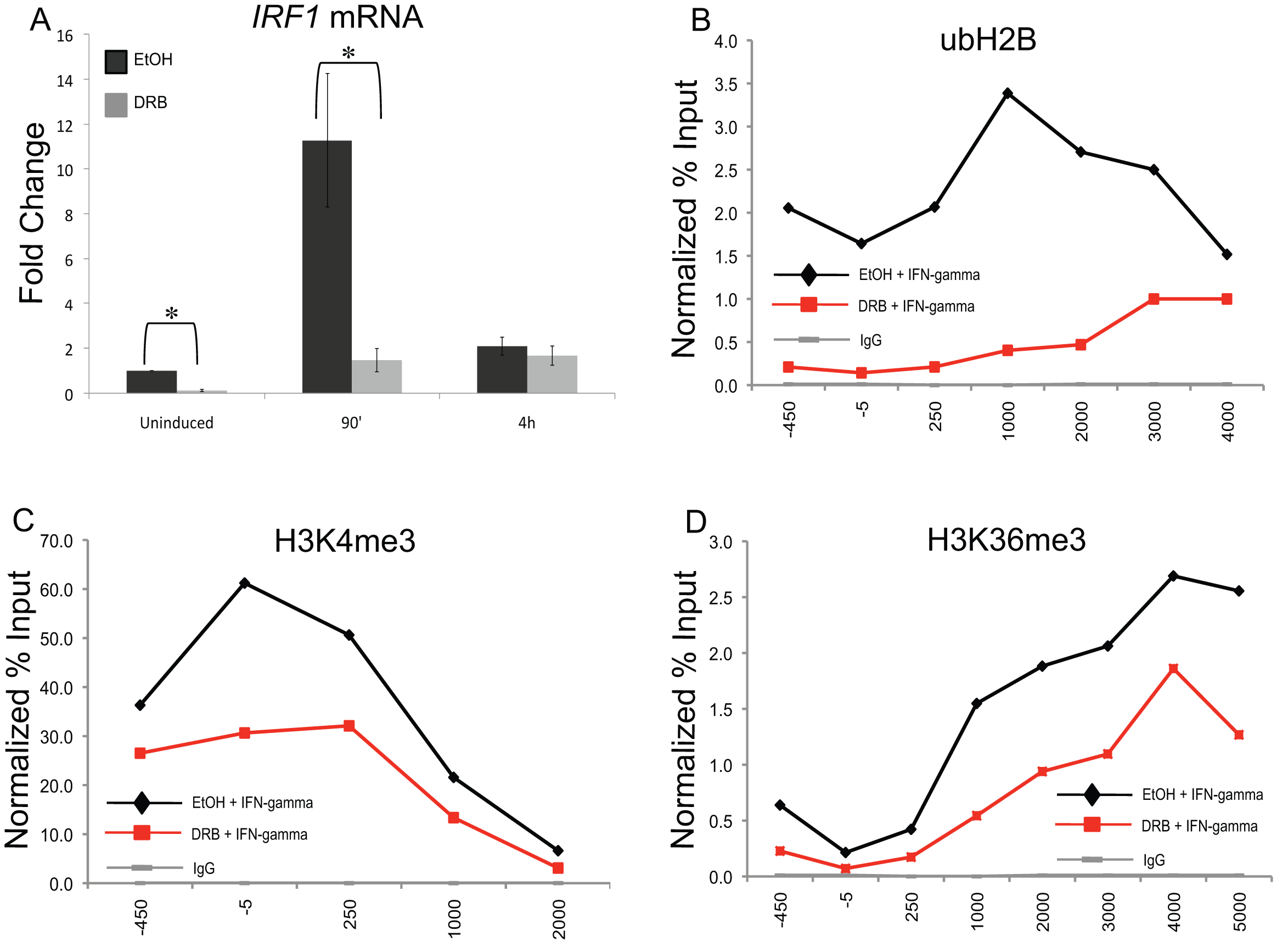

Supplement: Additional file 3 — 5,6-Dichlorobenzimidazole riboside (DRB) inhibition of transcription elongation prevents dynamic ubH2B, H3K4me3 and H3K36me3 during interferon regulatory factor 1 gene (IRF1) induction. (a) 2fTGH cells were treated with DRB (in ethanol, 25 mg/ml) for 10 min prior to induction with interferon (IFN)γ. Cells were collected and RT Q-PCR was performed to quantitate IRF1 expression relative to GAPDH and presented as fold change upon induction. Error bars represent standard error (n = 2). *P = 0.05. (b-d) 2fTGH cells were treated with DRB for 10 min prior to induction with IFNγ. ChIP assays with ubH2B, H3K4me3 and H3K36me3 antibodies were performed and real-time quantitative PCR (Q-PCR) quantified the precipitate yield, reported as percentage of input. [file 1756-8935-3-16-S3.PNG]
